# Experiences of Adults With Type 1 Diabetes Using Glucose Sensor–Based Mobile Technology for Glycemic Variability: Qualitative Study

**DOI:** 10.2196/14032

**Published:** 2019-07-08

**Authors:** Marilyn D Ritholz, Owen Henn, Astrid Atakov Castillo, Howard Wolpert, Stephanie Edwards, Lawrence Fisher, Elena Toschi

**Affiliations:** 1 Joslin Diabetes Center Boston, MA United States; 2 Department of Psychiatry Harvard Medical School Boston, MA United States; 3 Department of Medicine Harvard Medical School Boston, MA United States; 4 University of California, San Francisco San Francisco, CA United States

**Keywords:** diabetes mellitus, type 1, educational technology, blood glucose self-monitoring, qualitative research

## Abstract

**Background:**

Adults with type 1 diabetes (PWDs) face challenging self-management regimens including monitoring their glucose values multiple times a day to assist with achieving glycemic targets and reduce the risk of long-term diabetes complications. Recent advances in diabetes technology have reportedly improved glycemia, but little is known about how PWDs utilize mobile technology to make positive changes in their diabetes self-management.

**Objective:**

The aim of this qualitative study was to explore PWDs’ experiences using *Sugar Sleuth*, a glucose sensor–based mobile app and Web-based reporting system, integrated with the FreeStyle Libre glucose monitor that provides feedback about glycemic variability.

**Methods:**

We used a qualitative descriptive research design and conducted semistructured interviews with 10 PWDs (baseline mean glycated hemoglobin, HbA_1c_) 8.0%, (SD 0.45); 6 males and 4 females, aged 52 years (SD 15), type 1 diabetes (T1D) duration 31 years (SD 13), 40% (4/10, insulin pump) following a 14-week intervention during which they received clinical support and used *Sugar Sleuth* to evaluate and understand their glucose data. Audio-recorded interviews were transcribed, coded, and analyzed using thematic analysis and NVivo 11 (QSR International Pty Ltd).

**Results:**

A total of 4 main themes emerged from the data. Participants perceived *Sugar Sleuth* as an *Empowering Tool* that served to inform lifestyle choices and diabetes self-management tasks, promoted preemptive self-care actions, and improved discussions with clinicians. They also described *Sugar Sleuth* as providing a *Source of Psychosocial Support* and offering relief from worry, reducing glycemic uncertainty, and supporting positive feelings about everyday life with diabetes. Participants varied in their *Approaches to Glycemic Data:* 40% (4/10) described using *Sugar Sleuth* to review data, understand glycemic cause and effect, and plan for future self-care. On the contrary, 60% (6/10) were reluctant to review past data; they described receiving benefits from the immediate numbers and trend arrows, but the app still prompted them to enter in the suspected causes of glucose excursions within hours of their occurrence. Finally, only 2 participants voiced *Concerns About Use of Sugar Sleuth*; they perceived the app as sometimes too demanding of information or as not attuned to the socioeconomic backgrounds of PWDs from diverse populations.

**Conclusions:**

Results suggest that *Sugar Sleuth* can be an effective educational tool to enhance both patient-clinician collaboration and diabetes self-management. Findings also highlight the importance of exploring psychosocial and socioeconomic factors that may advance the understanding of PWDs’ individual differences when using glycemic technology and may promote the development of customized mobile tools to improve diabetes self-management.

## Introduction

### Background

Following the results of the Diabetes Control and Complications Trial [1] and the start of intensive insulin therapy, clinicians have attempted to help persons with type 1 diabetes (T1D) keep their glucose values as close to the normal range as possible to delay the onset and slow the progression of long-term diabetes complications such as retinopathy, renal disease, neuropathy, and heart disease. Monitoring glucose levels is essential for achieving target glycemia and avoiding hypoglycemia. Continuous Glucose Monitoring (CGM) is a recent glucose monitoring device that assists with these aims.

CGM has been shown to improve glycemia without an increase in hypoglycemia for adults with T1D (PWDs) who wear it most days [2-6]. In addition, studies have reported positive psychosocial changes such as decreased partners’ anxiety, vigilance and negative experiences surrounding hypoglycemia, and improved patients’ mood and general quality of life [7-8]. Similarly, a recent survey of 22,697 T1D Exchange Registry participants (aged 1-93 years) found that CGM usage increased from 7% in 2010 to 2012 to 30% in 2016 to 2018 and glycated hemoglobin (HbA_1c_) levels were lower in CGM users than nonusers [9]. Furthermore, technology improvements include an increased use of mobile health (mHealth) technology to support achieving optimal glycemia. However, more studies are needed to understand how mHealth devices influence diabetes self-management and improve glycemia [10]. Thus far, studies have mostly explored patient opinions on helpfulness and satisfaction with mHealth devices using quantitative self-report measures [11]. Qualitative studies can allow for a more in-depth understanding of how an intervention may individually affect PWDs and what to target for future interventions [10].

### Sugar Sleuth Technology

In our pilot intervention study, PWDs used a new glucose sensor–based tool, the *Sugar Sleuth* system, a specially designed interactive mobile app and Web-based reporting software integrated with the FreeStyle Libre, a glucose sensor (Abbott Diabetes Care) that provides feedback about glycemic variability to the PWD and the study investigators [12]. Importantly, glycemic variability is reported to limit the ability of PWDs to reach their HbA_1c_ targets without causing excessive hypoglycemia [13]. Furthermore, commercially available CGM devices inform the PWD when glucose levels are going up or down and acknowledge only patterns with no causes but *Sugar Sleuth* performs the unique task of asking why patterns are the way they are. In other words, this system was designed to identify the most prevalent causes of glucose excursions—highs, lows, and rapid rises—that contribute to variability and to provide reports in which the clinician and PWD can quickly understand the key problems that need to be addressed. Therefore, *Sugar Sleuth* is a unique strategy for helping people to improve their diabetes self-management because it is able to provide context or organization to PWDs’ out-of-range glucose levels, key barriers to target glucose levels, and actionable insights to perform. Thus, the aim of our study was to explore qualitatively PWDs’ experiences using this integrated *Sugar Sleuth* technology to better understand how their experiences affected their diabetes self-management.

## Methods

### Design and Ethics

We used a qualitative descriptive research design to obtain in-depth information about PWDs’ experiences using a mobile app for improving their diabetes self-management. We administered a one-on-one semistructured interview to a sample of PWDs naïve to CGM and following their participation in the *Sugar Sleuth* intervention at the Joslin Diabetes Center **,** which is an outpatient diabetes treatment and research center in the northeastern United States. The purpose of the intervention was to learn more about patient and clinician reactions to using a comprehensive mobile app linked to FreeStyle Libre. In short, participants attended 5 clinic visits over 14 weeks. Clinicians provided participants with the *Sugar Sleuth* system consisting of FreeStyle Libre, a wearable glucose sensor, and a mobile phone on which the *Sugar Sleuth* app was installed. Upon scanning the glucose sensor with the phone, the app displays the current glucose value as well as a trend arrow, which visually illustrates the direction and rate of change in glucose. In addition, the *Sugar Sleuth* app generates prompts for more information from the participant if a high, low, or rapid-rise episode is detected and provides a checklist of self-care issues that might have been the cause of the episode, for example, high carb meal or too much insulin. Thus, the app, Web-based report software, and glucose sensor provide an integrated, comprehensive system for detecting glycemic variability, specific problem identification, associated problem cause, plan for corrective action, and monitoring of intervention effects. Clinicians and participants collaboratively analyzed and reviewed collected data and devised specific self-care plans to address each event detected by Sugar Sleuth. Quantitative methods and results for this study were previously reported [12]. The Institutional Review Board at the Joslin Diabetes Center approved the protocol, and all participants provided written informed consent and received a small stipend.

### Study Participants

The study recruited 10 of the 30 participants who had completed the *Sugar Sleuth* intervention study for the qualitative interviews. Qualifications for the intervention study included T1D for at least 1 year, aged 25 to 75 years, treated with multiple daily injections or insulin pump, no previous use of CGM, not pregnant, no diagnosis of gastroparesis, no past bariatric surgery, and with HbA_1c_ between 7.5% and 9.5%. All 30 participants were contacted via email or by phone call and were asked to meet with the interviewer in the evenings or on weekends at the Joslin Diabetes Center. The first 10 participants who were able to meet with the interviewer at the available times were interviewed.

### Data Collection

A clinical psychologist (MDR), an experienced interviewer with extensive experience treating adult patients with T1D, conducted the qualitative interviews. These interviews asked participants open-ended questions about their experiences using the *Sugar Sleuth* technology and also used directive probes to elicit additional information and clarify questions (Textbox 1). Interviews lasted 30 to 60 min and were digitally recorded and transcribed.

**Sugar Sleuth semistructured interview questions.**

1. How did you expect the technology in this study to influence you as a person with diabetes?
  Probe: What were your expectations of the technology’s influence on your diabetes management?
  Probe: What were your expectations of the technology’s influence on your daily life?
  Probe: Were your expectations realized? If so, how? If not, how?
2. What was positive in your experience of using the technology in this study?
  Probe: How has the technology positively affected the way you manage your diabetes?
  Probe: How has the technology positively affected the way you think about your diabetes?
3. What was negative in your experience of using the technology in this study?
  Probe: How has the technology negatively affected the way you manage your diabetes?
  Probe: How has the technology negatively affected the way you think about your diabetes?
4. What would you change about the technology to better support your diabetes management and everyday life?
  Probe: How would these changes help you better manage your diabetes?
  Probe: How would these changes improve your life with diabetes?
5. What were your concerns about using the technology in this study?
  Probe: How do you feel about the amount of information the device supplies?
  Probe: How do you feel about the technology’s convenience/lack of convenience?
  Probe: How did you cope with these concerns?
6. How did you feel about using the device?
  Probe: What was most difficult and easiest about wearing the device?
  Probe: How often would you want to wear the device? Explain.
  Probe: How did you respond to the tracings?
  Probe: What do you think and feel when viewing the tracings?
  Probe: How do you utilize the tracings in your diabetes management?

### Data Analysis

The multidisciplinary research analysis team was diverse in terms of gender, disciplines, age, years of experience, and ethnic backgrounds. It included a woman senior clinical psychologist, a woman adult endocrinologist, a younger man researcher, and an experienced woman researcher**,** and 2 people with American English as a second language (ET and AAC). The team met over the course of 4 months to analyze data according to the principles of thematic analysis [14]. They independently read transcripts and coded the data by marking and categorizing key words and phrases and used an iterative approach whereby codes were continuously revised and refined throughout the analysis. Data analysis continued until data saturation for each theme occurred. After transcripts were coded and reviewed, one member of the research team (OH) entered the marked transcripts into NVivo 11 (QSR International Pty Ltd) to further organize and group codes into themes. The group then met to agree on the final themes and to select quotations that represented each theme. An audit trail tracked the decision-making process and supported the dependability (reliability) of the data.

## Results

A total of 10 PWDs participated in the interviews. There were no demographic differences between those participants who were interviewed and those who were not interviewed (Table 1).

**Table 1:** Characteristics of interviewed versus noninterviewed participants. Student *t* tests were used to examine differences between those interviewed and not interviewed.

Demographic characteristics		Semistructured interviewed (n=10)	Not interviewed (n=20)	*P* value
**Gender, n (%)**				**.32**
	Male	6 (60)	12 (60)	
	Female	4 (40)	8 (40)	
Age (years), mean (SD)		52 (15)	56 (14)	.53
**Ethnicity, n (%)**				**.02**
	Non-Hispanic	9 (90)	16 (80^a^)	
	Hispanic	1 (10)	1 (5^a^)	
Duration of type 1 diabetes (years), mean (SD)		31 (13)	33 (17)	.75
**Insulin use, n (%)**				**.80**
	Pump	4 (40)	9 (45)	
	Multiple daily injections	6 (60)	11 (55)	
HbA_1c_^b^ % baseline, mean (SD)		8.0 (0.45)	8.0 (0.60)	.78
HbA_1c_ % end of study, mean (SD)		7.6 (0.50)	7.5 (0.50)	.38

^a^A total of 15% (3/20) did not answer.

^b^HbA_1c_: glycated hemoglobin.

### Emergent Themes

Qualitative analysis revealed 4 main themes that described participants’ experiences with *Sugar Sleuth* and their diabetes self-management: *Empowering Tool, Source of Psychosocial Support, Approaches to Glycemic Data: Overview versus Narrow View, and Concerns About Use*. Transcript identifiers (age, sex, and years with T1D) are included with quotations.

### Empowering Tool

Participants described *Sugar Sleuth* as providing an empowering educational tool that informed their lifestyle choices and promoted their engagement in diabetes self-care tasks. *Sugar Sleuth* also fostered preemptive self-care actions as well as improved discussions with their clinicians. Furthermore, participants reported that *Sugar Sleuth* provided constant and immediate feedback that increased their understanding of the factors contributing to their glycemic excursions (variability) and how and when to address these excursions:

Well just seeing where my blood sugars were going, and being able to keep track of everything in one location, what I was eating, my activity level, um, my insulin dosages, and then being able to see snapshots of where you went low when you went for a 2 mile walk and just compare it to a day where I sat at my desk all day.... It really helped me to understand how to better adjust my insulin dosages, to better reflect, or to have better control and fewer fluctuations.

54-year-old woman, T1D for 34 years

...It’s one thing to hear ‘avoid being low it could kill you,’ which hadn’t been my experience with the first 40-50 years of management, but seeing it happen and nipping it in the bud, I could see a downward trend or a fast downward trend, I could catch it before it became an issue, and the same on the high ends. Yeah it was definitely good to avoid the extremes.

65-year-old man, T1D for 59 years

In terms of lifestyle choices and behaviors, participants reported that *Sugar Sleuth* increased their awareness and understanding about appropriate food choices and thereby contributed to changes in their eating behaviors:

It was probably making me more aware of what foods I was eating, what kind of effect it had on my body, where I sort of knew but didn’t pay much attention to it. But seeing concrete information with what I had entered into the system with the clinicians looking at it, and charting those highs and lows, it really gave me more of an understanding.

60-year-old man, T1D for 32 years

It gets me to look at more of what I eat, anything that makes me look at more of the nutritional factors on the back of the boxes or anything like that, that got me really to slow down, take a look at exactly what I’m eating and stuff like that, so that was a huge improvement.

40-year-old woman, T1D for 22 years

Finally, participants viewed *Sugar Sleuth* as empowering because it enhanced supportive and encouraging interaction between the patient and clinician and allowed the PWD to feel more actively engaged in their diabetes management:

And it’s also been really helpful when I have seen my doctor. Instead of just looking at a static list of blood sugars and insulin doses, to look at the graph and make adjustments. ...At my last visit... he (doctor) looked at the numbers first but then looked at the graph and then just said, “Actually I think you’re doing a great job because I’m not seeing the wild fluctuations that we would see before. There’s still some tweaking, but this is great information and you’re doing a great job.” And that’s the first time my doctor has ever said you’re doing a great job. ... I think it’s all because of just having access to more, more data points, more information.

54-year-old woman, T1D for 34 years

I loved the technology. I loved the fact that I was focusing on entering in all the foods that I was eating, and I loved the fact that I could talk with Joslin (clinicians), and they were talking about some of my highs, when they occurred, what I had eaten, and it really got me more on track.

60-year-old man, T1D for 23 years

### Source of Psychosocial Support

Participants described *Sugar Sleuth* as offering relief from worry and as helping them cope more effectively with T1D. They perceived the constant information that *Sugar Sleuth* provided as making for less glycemic uncertainty; they and others worried less about whether or not they were hypo- or hyperglycemic. These feelings contributed to participants feeling more positive about their everyday lives with diabetes:

And checking out this food and watching after the meal how’s it going; and going out for exercise, beginning middle and end because it’s there; I don’t have to stab myself, I can see it.... I could show it to my friends – “see I’m normal you don’t have to worry.” That was a relief to me and a relief to them.

65-year-old man, T1D for 59 years

It was a confirmation ... that I was in range more than I thought I was. And that was surprising because in the past when you only check your blood sugar every so many hours you get the feeling that you’re always up and down, up and down, which I am a lot, but I was in range in parts of the day much more than I thought. That was a good feeling. That was reinforcement.

76-year-old woman, T1D for 37 years

Furthermore, participants described how *Sugar Sleuth* made living with T1D more manageable and thus less burdensome. For example, *Sugar Sleuth’s* graphic information, unlike finger-stick numbers, made participants feel less powerless and more in charge of their T1D:

The graphical presentation—because up until then all I had was 4 data points for an entire day and I had no idea what was going in between—not no idea, but that isn’t what was really happening in real life. You get to see what is actually happening—it’s the truth or as close as we’re gonna get.

65-year-old man, T1D for 59 years

I think it [Sugar Sleuth] just made it [diabetes] um, easier to manage; easier isn’t really the right word, but it just, it just, it enhanced having it [diabetes]. I didn’t think about it more. I didn’t think about it less; it just made dealing with it [diabetes] more pleasant.

50-year-old woman, T1D for 28 years

### Approaches to Glycemic Data: Overview Versus Narrow View

A total of 4 participants described utilizing an overview approach to their glycemic data, which included actively reviewing graphs and app information to better understand cause and effect regarding their glycemia and to plan for future self-care:

I love the graphs, I love the longer time base thing. Day to day is one thing, but then you look at 2 weeks’ worth of data and you can see maybe I wasn’t as good as I thought and maybe I was better than I thought. The averaging and the probability-based parts of it were interesting.

64-year-old man, T1D for 25 years

In terms of the suspected causes and the list that it had, I think that’s something that’s definitely helpful and if you take the time to sit down and locate what’s consistently causing a rapid rise it helps you to figure that out and fix it going forward.

30-year-old man, T1D for 16 years

On the contrary, 6 participants described having a narrow view or focusing only on the immediate data and were reluctant to use the app or Web-based information to review data. However, they were still engaged with the app, that is, the app still prompted them to enter in the suspected causes of glucose excursions within hours of their occurrence. This narrow view suggests that the immediate information they received was sufficient, and they did not want or need to focus on past information and generally wanted to *move on*:

In real life, I’m probably not gonna get up every morning and look all night long and say, “oh look I went up, I went down...” It’s just not gonna happen...Maybe I don’t get the use of all the data but just that arrow going up and down and telling you what’s happening right now is enough to make you do stuff.

55-year-old woman, T1D for 47 years

Once the episode was over I wasn’t so much interested in hearing about it again. Maybe it was the ones that typically happened overnight when I wasn’t aware—if I went high overnight—that was last night, today is today—let’s move on.

65-year-old man, T1D for 59 years

### Concerns About Use

Only 2 participants reported negative perceptions about their use of the *Sugar Sleuth* system. They perceived the app as too demanding of information or as not attuned to the socioeconomic backgrounds of diverse populations. For example, one participant stated that the app’s demand for information did not always fit with where he was in his glycemia. He noted how difficult it was to answer questions about the causes of his hypoglycemia while he was in the midst of a hypoglycemia episode:

Like when I’m low I can’t really say like yeah I didn’t eat or I took too much insulin. Maybe like after I take care of the low then I can go back and say oh yeah this is why I was low.

28-year-old man, T1D for 19 years

The second participant observed that the app’s excessive demand for information did not fit well into the average person’s life:

Too much information. Like I said I did it just because I was in a study. So I did it. But your average Joe person is not gonna do that. People are just way too impatient; it’s just how it is. As sad as it is, that’s how it is.

40-year-old man, T1D for 22 years

Interestingly, this second participant was the only Hispanic person in the study. He voiced concern that it might be too difficult for PWDs from a lower socioeconomic or a different cultural background to adopt this technology because they do not have access to the same information or resources as those from the majority culture. He pointedly illustrated his concern when reporting how much it bothered him that he could not understand some of the choices offered by the app:

Who really uses this [technology] in the overall general public? If there was somebody that’s a severe diabetic and is in a low-income environment... they wouldn’t know what this is... I’m also talking about other religions, cultures and stuff like that Hispanics, Blacks...they would not understand what this is.... ...the options that they had were kind of, I wasn’t really, didn’t really know exactly what they meant. So to me that was the thing that really got me.”

40-year-old man, T1D for 22 years

## Discussion

### Principal Findings

In this qualitative study, participants described how the integrated *Sugar Sleuth* system empowered them as PWDs to make more informed food choices, to preemptively avoid glycemic excursions, to improve problem-solving discussions with their clinicians, and to have a greater awareness of cause and effect surrounding their glycemia. Furthermore, they described this system as providing comfort and reassurance and thereby serving as a source of psychosocial support. In addition, the results of the previously published quantitative study indicated a significant average reduction in HbA_1c_ from baseline mean of 8.0% (SD 0.10%) to final mean of 7.5% (SD 0.09%; *P*<.01) and in mean daily carbohydrate intake from baseline 235 (SD 21) grams to final 192 (SD 26) grams (*P*=.05), which reflect the empowering qualitative experiences that participants described [12]. However, our study also found variability in PWDs’ approaches to the use of glycemic information. All participants used the glucose information to improve their diabetes self-management but some benefited from the immediate feedback whereas others preferred retrospective data review.

Of note, 40% (4/10) of participants utilized retrospective review to help manage their diabetes both in the present and in the future. Alternatively, 60% (6/10) did not review their data and described finding it useful to focus solely on immediate numbers and trend arrows. This finding is of interest in the world of diabetes treatment where retrospective review of Glucose Pattern Management (GPM) is often described as an essential ingredient for improving diabetes self-management [15]. The goal of GPM is to reduce the frequency of undesirable glycemic patterns to improve clinical outcomes and to lighten the burden of diabetes management for PWD [16], but studies have found that most CGM users never download data from their devices [17]. In fact, we do not really know if CGM users mostly rely on their current glucose value, glucose profile over the past few hours, or rate of change (trend) arrows to make insulin adjustment decisions [18]. Furthermore, although the recent study of T1D Exchange participants found increased use of CGM among its 22,697 registry participants, the downloading and retrospective review of the CGM data as part of diabetes self-management had not increased and there was no indication that HbA_1c_ levels in the registry as a whole have improved over the 5-year period despite this increased use of CGM [9]. Thus, there appears to be a real need for increased education about how to utilize the CGM data offered to PWDs.

Moreover, the one Hispanic participant in our study wondered if *Sugar Sleuth* was appropriate for PWDs from a low socioeconomic or different racial or cultural background. The participant’s comment suggests that *Sugar Sleuth* may require increased adaptation to the educational and cultural needs of diverse populations. In fact, in a recent review of studies on mHealth technology and underserved populations, the authors strongly note the importance of tailoring mHealth interventions in a culturally competent manner and of instituting curriculums at literacy levels appropriate to target populations to optimally utilize this technology [19].

Finally, our results suggest that the *Sugar Sleuth* system taught participants a meaningful way to use their glycemic data for diabetes self-management by helping them identify the causes of their glycemic excursions and guiding them to make appropriate diabetes self-management decisions. Interestingly, another recent study suggests that the discovery of cause and effect in diabetes for persons with type 2 diabetes can help improve their self-management strategies and that self-monitoring data can initiate personal discovery that may lead to sustainable behavior changes [20]. Although the results of our study support this finding, efforts are called for to further document this finding for T1D adults. Importantly, 2 participants also perceived the system as too demanding for information or as not attuned to lower socioeconomic and different cultural groups and addressing these perceptions may enhance the use of *Sugar Sleuth*. In addition, research needs to explore how technologies can be used to help PWDs solve specific diabetes self-management problems [21] and how to improve the integration of mobile technology into everyday life.

### Limitations

Study limitations include the use of a small, homogenous (eg, English-speaking, 90% non-Hispanic, with long-duration T1D) sample from a specialty diabetes clinic in the northeastern United States. Furthermore, the intervention is a short, single-arm, single-center study. Therefore, results cannot be attributed to one of the 3 interventions used: Sugar Sleuth app, educational module, and flash glucose sensor. Studies to compare the benefit and long-term sustainability of results for the Sugar Sleuth app on its own versus all 3 combined components are needed. In addition, this qualitative study did not use an iterative data collection approach, which limits the inclusion of additional participants who might have expanded the description of the experiences studied. Future studies should include a larger sample using iterative data collection and data saturation so that the research question is more fully explored. In addition, our participants agreed to participate in this interview study and thus may have been more willing or motivated to perceive the benefits of *Sugar Sleuth* for their diabetes self-management than those who did not agree to participate. Finally, self-reported data are vulnerable to social desirability bias.

### Conclusions and Implications

The findings of this qualitative study have important implications for clinical care. Primarily, they suggest that a glucose sensor–based mobile technology can be an effective educational tool to enhance both patient-clinician collaboration and diabetes self-management. These results suggest the clinical usefulness for evaluating the experience of diabetes mobile technology in a larger, more diverse population, given the demographic characteristics of our sample. Results also highlight the importance of exploring psychosocial factors such as cognitive processing, decision making, diabetes distress, depression, and anxiety that may advance the understanding of individual differences in PWDs’ use of mobile technology to improve diabetes self-management. As the world of diabetes self-management moves increasingly toward mobile technology, diabetes researchers and clinicians need to understand better how each PWD’s cognitive and emotional attributes influence his/her ability to use glycemic information for diabetes self-management. Importantly, this understanding may help avoid the too often made assumption in clinical care that one size fits all. Thus, customizing technological devices to meet individual cognitive and emotional needs and characteristics may provide more personalized mobile education tools for optimal diabetes management and allow not only for improved glycemia but also for improved quality of life for PWDs.

## Abbreviations

HbA_1c_: glycated hemoglobin
CGM: Continuous Glucose Monitor
GPM: Glucose Pattern Management
mHealth: mobile health
PWD: adult with type 1 diabetes
T1D: type 1 diabetes
